# Direct-Contact Low-Frequency Ultrasound Clearance of Biofilm From Metallic Implant Materials

**Published:** 2017-03-29

**Authors:** Mark S. Granick, Chaitra Paribathan, Mayilvahanan Shanmugam, Narayanan Ramasubbu

**Affiliations:** ^a^Department of Surgery, Division of Plastic Surgery, Rutgers New Jersey Medical School, Newark; ^b^Department of Oral Biology, Rutgers New Jersey Dental School, Newark

**Keywords:** biofilm, debridement, ultrasound, infected implant, chronic wound

## Abstract

**Introduction:** Biofilms are recognized as a significant deterrent to wound healing and to the management of exposed or infected surgical implants. Biofilms can be disrupted by a variety of enzymatic and mechanical interventions. This experiment was designed to determine whether direct-contact low-frequency ultrasound has the ability to clear biofilms and what then happens to the released bacteria. **Methods:** *Staphylococcus epidermidis* biofilm was grown on the surfaces of metallic discs composed of titanium and stainless steel, comparable with the alloys used in surgical implants. The discs were treated with a control of irrigation and no ultrasound, followed by the ultrasound for a 10 second of exposure at a mid-level power setting. The irrigation materials used was either normal saline or hypochlorous acid. The effluent was cultured to determine colony-forming units, and the discs were stained with crystal violet to determine whether there was a residual biofilm. **Results:** The biofilm was cleared completely from all discs when treated with direct-contact low-frequency ultrasound. However, the released bacteria were viable and could be cultured from the effluent when saline was used as the irrigation medium. When hypochlorous acid was used as the irrigation medium, there was complete killing of all planktonic bacteria. **Conclusion:** Direct-contact low-frequency ultrasound is effective when used to clear biofilms from metallic implant materials. By using hypochlorous acid as the irrigant during treatment, all of the bacteria released from the biofilm were killed as well. The implications for clinical application are important and need to be independently studied.

Infected orthopedic implants are a source of considerable patient morbidity as well as a critical cost factor. A direct-contact low-frequency ultrasound (DCLFU) device was introduced for the purpose of wound debridement. This consists of a handpiece that delivers low-frequency ultrasound (20 kHz) and uses an irrigant for energy transfer to the tissues. The tissue interaction is facilitated by bubble cavitation and acoustic streaming to remove necrotic material. A recent observation by an orthopedic team that implant-associated wounds debrided with this instrument clinically had fewer postoperative implant infections led us to investigate this observation in a laboratory setting. Ultrasound has long been known to clear biofilms off of metal pipes and other surfaces. This study was planned to determine whether energy delivered by the DCLFU debridement device is sufficient to remove biofilms from metallic implant surfaces.

## METHODS

**Figure 1:**
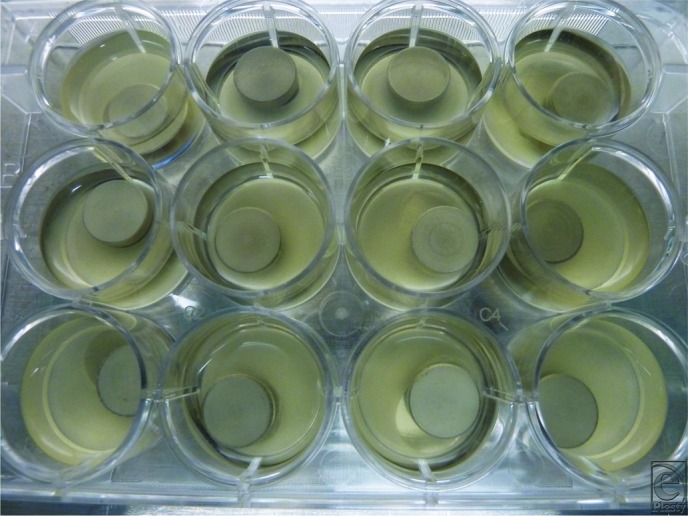
Biofilm grown on titanium alloy and stainless steel discs.

**Figure 2:**
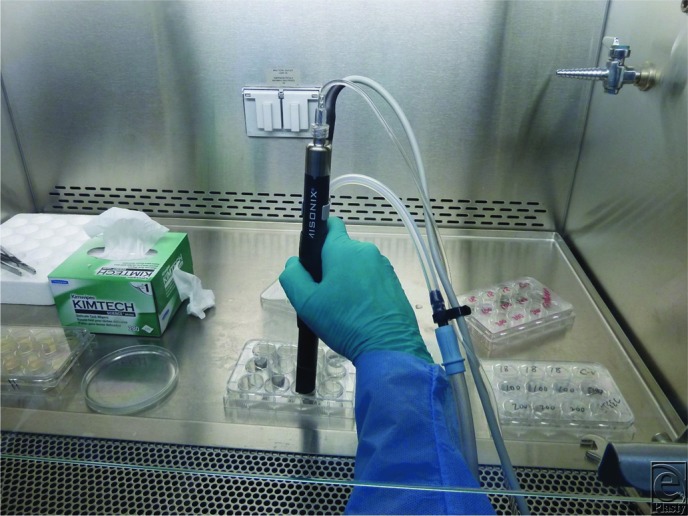
Experimental setup. The study was conducted under a hood. The direct-contact low-frequency ultrasound device was placed 2 mm above each disc for 10 seconds.

*Staphylococcus epidermidis* (RP62A) was used to grow the biofilm on 1-cm discs of titanium alloy and stainless steel, comparable with implant metal alloys. The discs were incubated in 2-cm diameter plastic wells (Fig 1). Control trials were performed by irrigating biofilm discs through the handpiece with the DCLFU device off. Control trials at a 20% flow rate were performed for both saline irrigation and hypochlorous acid (HCA) irrigation. In the experimental trials, the discs were treated with the DCLFU device wide hatch probe for 10 seconds, placed 2 mm above the discs, at 113-μm power without suction (Fig 2). Each trial consisted of 10 treated discs, 5 of titanium and 5 of stainless steel. The effluent was immediately plated onto blood agar Petri dishes, incubated, and counted at 24 hours. The discs were rinsed with phosphate buffered saline and then stained with crystal violet to determine whether there were residual bacteria present.

## RESULTS

**Figure 3:**
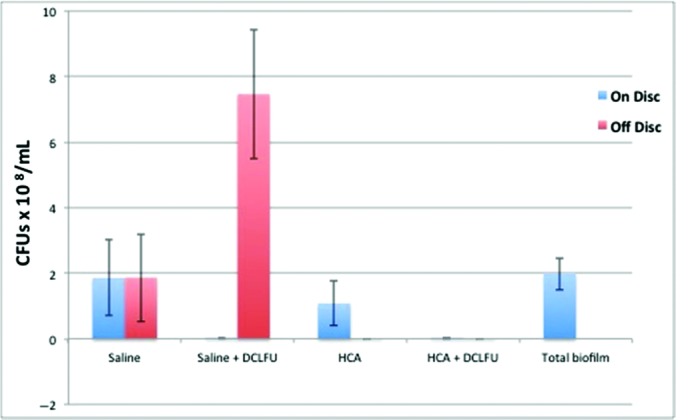
The data consist of controls of saline and HCA. The biofilm on the disc is colored blue and the effluent is colored pink. CFUs are assessed. The biofilm was removed by DCLFU in all cases. However, saline irrigation left large numbers of viable planktonic bacteria in the effluent. When HCA was the irrigant, there was complete killing of planktonic bacteria. HCA indicates hypochlorous acid; CFU, colony-forming unit; and DCLFU, direct-contact low-frequency ultrasound.

**Figure 4:**
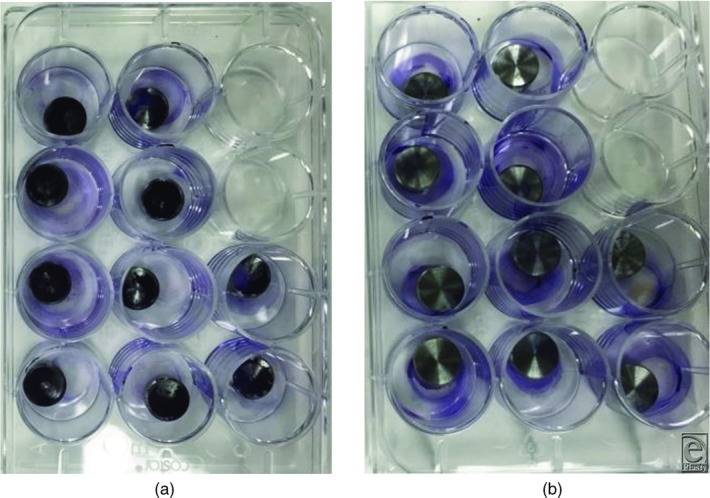
Crystal violet is a stain picked up by the biofilm. (a) Crystal violet–stained discs in controls. (b) Crystal violet completely removed when direct-contact low-frequency ultrasound was used.

**Figure 5:**
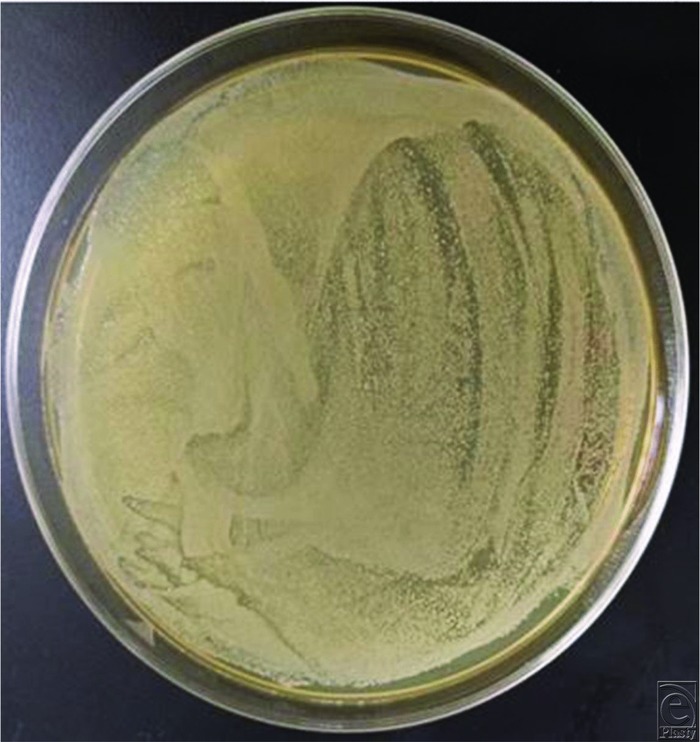
A lawn of bacteria was cultured from the effluent when saline was used as an irrigant.

The results are depicted in Figure 3. In the control study with a rinse of saline and with HCA, the bacterial counts in the effluent are minimal. When the ultrasound is turned on and the discs are treated, both the stainless steel and titanium discs responded similarly. All of the visible biofilm was removed. Crystal violet staining confirmed this gross observation (Figs 4*a* and 4*b*). The residual effluent, however, in the saline trial demonstrated high levels of viable bacterial cells. The growth plates presented a “lawn” of bacteria (Fig 5). In the HCA trial, there was no growth of bacteria in the effluent.

## DISCUSSION

Biofilms are a bacterial adaptation to environmental threats. Free-floating planktonic bacteria of many different species have the ability to aggregate and synthesize a polymeric matrix that attaches to a tissue or other surfaces. Biofilms are notoriously difficult to clinically control.

Ultrasound is a sound or other vibration with a frequency above the human hearing range. Ultrasound has been long been known to disaggregate biofilms^1^^,^^2^ and to similarly act as a biocide.^3^ Ultrasound biocidal activity occurs as a result of acoustic microstreaming and bubble cavitation. When the vibrations of the ultrasound occur in a liquid medium, gas bubbles occur at the nadir of the vibratory wave and compress at the apex. As the bubble enlarges through successive wave forms, it ultimately collapses on itself, releasing considerable energy, which can damage bacterial cell walls. Microstreaming occurs when the vibrations set up rapidly expanding and collapsing bubbles. The surrounding fluid develops a powerful but microscopic turbulence that imparts mechanical energy on targets.

Ultrasound is utilized in a wide range of medical instrumentation. A DCLFU device is available for wound debridement. The machine has a small power box and is connected to a handpiece with a foot pedal control. The handpiece has interchangeable probes that amplify and transmit ultrasound at 20-kHz frequency. A fluid interface is necessary to facilitate energy transmission to the tissues. Fluid is introduced through a small hole at the tip of the probe. The energy turns it into a mist. To control dispersal, a vacuum sheath surrounds the tip and removes the mist as well as some of the tissue debris from the operative field.

Two important findings led to this study. The first is that a number of orthopedists anecdotally reported that utilizing the DCLFU device in implant-related wounds facilitated implant salvage. The next derives from the introduction of the vacuum sheath to the handpiece. Effluent was collected in a line trap and analyzed. The fluid contained viable bacteria.^4^ Clearly, the ultrasonic energy delivered to the wound was not completely bactericidal. Although research has previously demonstrated that ultrasound delivered to the biofilm containing titanium and stainless steel implant materials disrupts the biofilm,^5^ it has not been demonstrated for the frequency, power, contact time, and configuration of the DCLFU device.

The study demonstrates that at the clinical utilized power settings and time of contact, the DCLFU apparatus disaggregates *S epidermidis* biofilm from the surfaces of titanium and stainless steel discs. When the irrigating solution is saline, viable planktonic bacteria appear in the effluent. However, the addition of HCA eliminates most if not all of the posttreatment bacterial contamination. These findings are consistent with a clinical study by Hiebert and Robson,^6^ in which wound debridement was performed with DCLFU using either HCA or saline irrigation. The immediate posttreatment tissue biopsies had similar bacterial loads, but after 4 days, the saline group had increased to 10^5^ whereas the HCA group remained at 10^2^. Presumably, the presence of HCA in the wound killed the planktonic bacteria remaining on the wound surface after debridement.

The findings of this study have powerful clinical implications. The immediate implication is that when using DCLFU debridement to optimize bacterial destruction following biofilm disaggregation, HCA is the irrigating fluid of choice. The other interesting finding is that at the moderate operating settings of the DCLFU device in this study, biofilms can be removed from implant materials by using the instrument adjacent to the implant. Periprosthetic joint infection (PJI) is the major complication of orthopedic joint implants.^6^^-^8 The majority (80%-90%) of PJIs are due to *Staphylococcus* sp. PJI leads to severe morbidity, multiple hospitalizations, prolonged medical and surgical treatment, and disruption of activities of daily living. It is similarly a drain on medical resources, costing well over $100,000 per infected joint. The incidence of PJIs in primary knee replacements is 1.3% and in primary hip replacements is 1.6%. Attempts to reduce infection rates have included all sorts of perioperative interventions, antibiotic administration, and altering the prosthetic surface to make it less hospitable to biofilms.^7^^-^9 The possibility of introducing topical DCLFU to disrupt biofilms in the wound and on the prosthesis, augmented with HCA biocidal activity, is promising. Our group is currently investigating this possibility in an in vivo model.

## CONCLUSION

This experiment demonstrates that the DCLFU device has the ability to clear *S epidermidis* biofilms off of metallic implant material. At the moderate power levels and brief exposure time used in this study, the bacteria are converted to the planktonic state with biofilm dispersion. Using HCA as the mechanical irrigant facilitates killing of the planktonic bacteria that are released. This has important clinical implications for the treatment of wounds in general and particularly wounds harboring exposed implant material. At least one published clinical study has supported this finding.^6^ Additionally, the parameters of biocidal activity of the DCLFU with regard to power levels and time of exposure need to be determined.

## DISCLOSURE

Dr. Granick is a medical and research consultant for Misonix, Inc.

## References

[1] Zips A, Schaule G, Flemming HC (2009). Ultrasound as a means of detaching biofilms. J Bioadhesion Biofilm Res.

[2] Bott TR (2000). Biofouling control with ultrasound. Heat Transfer Eng.

[3] Bott TR, Tianqing L (2004). Ultrasound enhancement of biocide efficiency. Ultrason Sonochem.

[4] Abolghasemi D, Baruch M, Granick MS

[5] Bjerkan G, Witso E, Bergh K (2009). Sonication is superior to scraping for retrieval of bacteria in biofilm on titanium and steel surfaces in vitro. Acta Orthop.

[6] Hiebert JM, Robson MC (2016). The immediate and delayed post-debridement effects on tissue bacterial wound counts of hypochlorous acid versus saline irrigation in chronic wounds. ePlasty.

[7] Gutowski CJ, Chen AF, Parvizi J., Kendoff K, Morgan-Jones R, Haddad F (2016). The incidence and socioeconomic Impact of periprosthetic joint infection: United States perspective. Periprosthetic Joint Infection.

[8] Montanaro L, Speziale P, Compoccia D (2011). Scenery of *Staphylococcus* implant infections in orthopedics. Future Microbiol.

[9] Drago L, Boot W, Dimas K (2014). Does implant coating with antibacterial-loaded hydrogel reduce bacterial colonization and biofilm formation in vitro?. Clin Orthop Relat Res..

